# Resveratrol Activates Autophagy via the AKT/mTOR Signaling Pathway to Improve Cognitive Dysfunction in Rats With Chronic Cerebral Hypoperfusion

**DOI:** 10.3389/fnins.2019.00859

**Published:** 2019-08-20

**Authors:** Nan Wang, Jinting He, Chengliang Pan, Jiaoqi Wang, Ming Ma, Xinxiu Shi, Zhongxin Xu

**Affiliations:** ^1^Department of Neurology, China-Japan Union Hospital of Jilin University, Changchun, China; ^2^College of Clinical Medicine, Jilin University, Changchun, China

**Keywords:** resveratrol, AKT, mTOR, cognitive dysfunction, chronic cerebral hypoperfusion

## Abstract

Chronic cerebral hypoperfusion (CCH) is a main cause of vascular dementia and is also an etiological factor of neurological diseases and mental disorders. However, few treatments are available for CCH, and new medications are needed. In the present study, we employed a rat model of CCH that was based on bilateral common carotid artery occlusion and investigated the therapeutic effects of resveratrol and its detailed mechanism of action. We evaluated neurological deficit scores and performed the Morris water maze test, hematoxylin and eosin staining, TUNEL staining, enzyme-linked immunosorbent assays, and Western blot. Resveratrol reduced neurological deficit scores in CCH rats and reduced pathological damage in the frontal cortex and hippocampus. Resveratrol activated autophagy and inhibited the expression of AKT/mechanistic target of rapamycin (mTOR) signaling pathway-related proteins. Treatment with a phosphoinositide-3 kinase inhibitor reversed the protective effect of resveratrol. These findings suggest that resveratrol improves cognitive function in a rat model of CCH and reduces oxidative stress-induced neuronal damage in the frontal cortex and hippocampus by activating autophagy and inhibiting neuronal apoptosis. These effects may be regulated by the AKT/mTOR signaling pathway.

## Introduction

Chronic cerebral hypoperfusion (CCH) can contribute to the development of various neurological diseases and mental disorders, including vascular dementia, Alzheimer’s disease, and Binswanger disease (Duncombe et al., 2017; Feng et al., 2018). Early stage CCH is mainly characterized by cognitive impairment that is followed by biological changes, such as energy metabolism disorders, abnormal neuronal electrical activity, oxidative stress, glial cell activation, and inflammatory factor release. These changes can lead to abnormal brain structure and function, including neuronal damage and degeneration of the frontal cortex and hippocampus, resulting in cognitive impairment (Du et al., 2017). One main factor that is involved in such damage is the transient expression of reactive oxygen species (ROS) that are induced by lower cerebral blood flow. Excessive ROS and free radicals can cause the apoptosis of nerve cells and astrocytes, leading to permanent nerve damage (Su et al., 2017). This process is associated with oxidative stress and mitochondrial dysfunction (Panchal and Tiwari, 2018).

In states of oxidative stress, cells undergo autophagy to remove damaged mitochondria, the endoplasmic reticulum, and proteins to slow the cell death process (Filomeni et al., 2015). Autophagy is a phenomenon of “self-eating” to recirculate molecules within cells. Impairments in autophagy are seen in many diseases, such as neurodegenerative diseases, insulin resistance, and immunity (Deretic et al., 2013; Moloudizargari et al., 2017; Tan et al., 2018). Previous studies have found that autophagy is initiated by inhibition of the rapamycin receptor mechanistic/mammalian target of rapamycin (mTOR), which is involved in various physiological and pathological cell processes and regulates cell growth, proliferation, differentiation, and autophagy by affecting protein synthesis (Dunlop and Tee, 2014; Kim and Guan, 2015; Munson and Ganley, 2015). In Alzheimer’s disease, low mTOR levels in peripheral blood lymphocytes may be related to disease progression. The inhibition of mTOR activity may impair memory consolidation, whereas activation of the AKT pathway may prevent the toxic effects of amyloid β (O’Neill, 2013). In epilepsy models, mTOR inhibition has been shown to reduce neuronal death, nerve regeneration, and the development of spontaneous epilepsy (Romero-Leguizamon et al., 2016). Long-lasting high levels of ROS can lead to excessive AKT/mTOR-regulated autophagy, transforming this normally protective pathway into an apoptosis-inducing mechanism (Wang X. et al., 2018).

Resveratrol is a natural polyphenolic phytoalexin with a range of protective physiological functions, including antioxidative and anti-inflammatory effects. It is used for the management of diabetes, neuroprotection and myocardium protection. Several studies reported that resveratrol induced autophagy (Bagul and Banerjee, 2015; Wan et al., 2016; Ling et al., 2017; Chen et al., 2018). Huang et al. (2016) found that resveratrol alleviated the hydrogen peroxide-mediated apoptosis of cardiomyocytes by regulating autophagic flow. Resveratrol was shown to reduce oxidative stress-induced brain damage in an Alzheimer’s disease model by inducing autophagy to reduce oxidative stress (Wang H. et al., 2018). Resveratrol was reported to alleviate cerebral ischemia/reperfusion injury in rats by inhibiting NLRP3 inflammasome activation through Sirt1-dependent autophagy induction (He Q. et al., 2017), but the effect of resveratrol on cerebral ischemia/reperfusion injury that is related to the AKT/mTOR pathway has not been reported. Resveratrol has a close relationship with AKT/mTOR. Resveratrol was shown to inhibit the phosphoinositide-3 kinase (PI3K)/AKT pathway to inhibit the proliferation and migration of hepatocellular carcinoma cells (Chai et al., 2017). Resveratrol was also reported to rescue hyperglycemia-induced endothelial dysfunction via the activation of AKT (Li et al., 2017). We hypothesized that resveratrol would ameliorate cognitive dysfunction that is caused by chronic cerebral ischemia, likely by the activation of autophagy through the AKT/mTOR signaling pathway. To test this hypothesis, we established a rat model of CCH that was induced by bilateral common carotid artery occlusion (BCCAO). The rats were treated with resveratrol, and we evaluated cognitive function and brain tissue apoptosis and autophagy. We investigated the possible involvement of the ATK/mTOR signaling pathway in the mechanism of action of resveratrol.

## Materials and Methods

### Laboratory Animals and Experimental Groups

A total of 96 male Sprague-Dawley rats, weighing 260–300 g, were purchased from the Beijing Vital River Laboratory Animal Technology Co., Ltd. The animal experiments were performed in the experimental animal department of China Medical University [experimental animal license no. SYXK (Liao) 20150001] with a 12 h/12 h light/dark cycle, controlled temperature (22°C ± 3°C), and controlled humidity (60% ± 5%). The animals had *ad libitum* access to food and water. The study was approved by the Experimental Animal Welfare and Ethics Committee of China Medical University (IACUC no. 2018097).

The rats were randomly divided into four groups using the random number table method (*n* = 24/group). The groups received the following treatments daily for 9 weeks: sham surgery and vehicle treatment (Sham group), CCH surgery and no treatment (CCH group), CCH surgery and resveratrol treatment (catalog no. R8350, Solarbio, Beijing, China; Res group), and CCH surgery and treatment with both resveratrol and the PI3K inhibitor LY294002 (PI3K group). After CCH was successfully established, determined by evaluating the rats in the CCH group relative to the Sham group, the Sham group was not used further.

### Establishment of Chronic Cerebral Hypoperfusion Model

**FIGURE 1:**
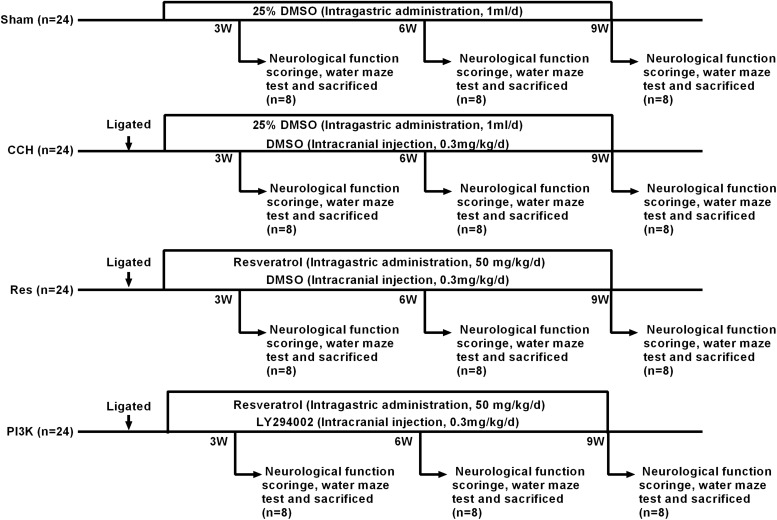
Schematic diagram of the rat groups and treatment regimens. Because the scores were zero in the Sham group, there is no bar-plot for this group.

The animals in each group were fasted for 12 h with water that was provided before surgery. The rats were anesthetized by an intraperitoneal injection of 2% sodium pentobarbital (3 mg/kg) and placed in the supine position. The skin on the neck was prepared, and a midline incision was made to separate the bilateral common carotid arteries and vagus nerve. In the groups that were subjected to CCH, the bilateral common carotid arteries were permanently ligated at the distal end of the telecentric end with No. 4 surgical thread. The incision was then closed by layer-by-layer sutures. Gentamicin was injected subcutaneously to prevent infection, and the rats were returned to their home cages for feeding. The Res group was intragastrically administered resveratrol (50 mg/kg/day) after surgery (Fang et al., 2018). After anesthesia with sodium pentobarbital in the PI3K group, a vertical incision was made along the midline to separate the fascia of the skull surface to fully expose the skull surface, which was then disinfected with hydrogen peroxide. The coordinates of the puncture site were 1.0 mm anterior/posterior and 1.5 mm from the center line, and the puncture site was marked with a marker. A dental drill was used to drill into the skull, exposing the dura mater. A stainless-steel injection catheter was then inserted (0.64 mm outer diameter) vertically into the puncture site, and gel was used on the surface of the skull to close the skin. The PI3K inhibitor LY294002 (Sigma-Aldrich, St. Louis, MO, United States; Shen et al., 2019; Zhan et al., 2019) was dissolved in dimethylsulfoxide (DMSO) at a concentration of 0.3 mg/ml and intracranially injected at a dose of 0.3 mg/kg at the beginning of hypoperfusion. In the Sham group, the bilateral common carotid arteries were separated from the nerves but not ligated, and 1 ml of 25% DMSO was intragastrically administered daily. Eight rats from each group were used for neurological function scoring and the Morris water maze test 3, 6, and 9 weeks after surgery. After the experiment, brain tissue samples were harvested. A portion was fixed in 4% paraformaldehyde, and the remainder was stored in a −80°C freezer. Blood was collected from the posterior saphenous vein, centrifuged to separate serum, and stored at −80°C (Figure 1).

### Detecting Behavioral Impairment in Rats Based on Neurological Deficit Scores

We used the Bederson neurological deficit scoring method (Desland et al., 2014). Symptoms in each group were observed, neurological deficits were recorded, and neurological function was scored. The scoring criteria were the following: 0 (no symptoms of nerve damage), 1 (contralateral forelimb cannot be fully extended when lifting the tail), 2 (the rats turn to the temporal side while walking), 3 (the rats fall to the opposite side of the lesion), and 4 (the rats cannot walk spontaneously).

### Testing Cognitive Function in Rats in the Morris Water Maze

Spatial memory function was assessed using the Morris water maze (Institute of Medicine, Chinese Academy of Medical Sciences, Beijing, China). The time spent locating the platform was recorded. The rats were placed in the water from four water inlet points on the pool wall twice daily for 5 days. The latency to find and climb on the platform (escape latency) in a 3 min session and the swimming path during this period (swimming distance) were recorded. The escape latency was used as a memory score for each rat, and average scores were calculated for four consecutive days. Afterward, the platform was removed, a water inlet point was selected to place the rat in the pool, and the number of times the rat crossed the original platform position during a 3 min session was recorded.

### Pathological Changes in the Frontal Cortex and Hippocampus Detected by Hematoxylin and Eosin Staining

Hematoxylin and eosin (HE) staining was used to evaluate neuronal damage in the frontal cortex and hippocampus. After behavioral testing, the rats were euthanized by deep anesthesia and bloodletting. Brain tissue was quickly placed on ice, and longitudinal sections of the brain were fixed with 4% paraformaldehyde. The H&E staining kit was purchased from Beyotime (Jiangsu, China), and the experimental procedure was performed according to the manufacturer’s instructions.

### Apoptosis in the Frontal Cortex and Hippocampus Detected by TUNEL Staining

Neuronal apoptosis in the frontal cortex and hippocampus was examined using a TUNEL staining kit (catalog no. 11684817910, Roche, Basel, Switzerland) according to the manufacturer’s instructions. Samples of the hippocampus and cerebral cortex were collected, fixed in paraformaldehyde, routinely dehydrated, paraffin embedding, and sectioned. Based on the number of slides and size of the tissue samples, the appropriate amounts of reagent 1 (TdT) and reagent 2 (dUTP) in the TUNEL kit were mixed in a 1:9 ratio and then added to the tissues. The sections were placed in a humidified 37°C incubator for 2 h and then evenly covered and blocked with 3% bovine serum albumin at room temperature for 30 min. Biotinylated nucleotides and terminal deoxynucleotidyl transferase were added to the sections (1:500 dilution) and incubated overnight at 4°C. The sections were then washed with phosphate-buffered saline (PBS) and covered with horseradish peroxidase (HRP)-labeled streptavidin in the dark for 50 min. DAPI staining solution was added, and the mixture was incubated at room temperature for 10 min in a darkroom, sealed with an anti-fluorescence quenching tablet, and photographed under a fluorescence microscope.

### Enzyme-Linked Immunosorbent Assay

The oxidative stress markers superoxide dismutase (SOD; catalog no. SES134Ra, USCN, Wuhan, China), malondialdehyde (MDA; catalog no. CEA597Ge, USCN), and reduced glutathione (GSH; catalog no. CEA294Ge, USCN) and brain damage markers S-100β (catalog no. SEA012Ra, USCN) and neuron-specific enolase (NSE; catalog no. SEA537Ra, USCN) were detected by enzyme-linked immunosorbent assay (ELISA) kits in brain tissues according to the manufacturer’s instructions.

### Immunofluorescence Assay

The immunofluorescence assay (IFA) was performed as previously described (Jing et al., 2015; Xiong et al., 2017). Briefly, sections were processed with relevant primary antibodies, including Bax (diluted 1:50, catalog no. ab32503, Abcam, Cambridge, United Kingdom), Bcl2 (diluted 1:50, catalog no. ab32124, Abcam, Cambridge, United Kingdom), cleaved caspase-3 (diluted 1:50, catalog no. ab13847, Abcam, Cambridge, United Kingdom), LC3B (diluted 1:50, catalog no. ab48394, Abcam, Cambridge, United Kingdom), Beclin 1 (diluted 1:50, catalog no. ab207612, Abcam, Cambridge, United Kingdom), mTOR (diluted 1:50, catalog no. ab2732, Abcam, Cambridge, United Kingdom), AKT (diluted 1:50, catalog no. ab8805, Abcam, Cambridge, United Kingdom), S6K1 (diluted 1:50, catalog no. ab32529, Abcam, Cambridge, United Kingdom), and 4E-BP1 (diluted 1:50, catalog no. ab2606, Abcam, Cambridge, United Kingdom). The sections were immersed in blocking solution and then incubated with fluorescent isothiocyanate-labeled secondary antibodies for 30 min at 37°C. After washing with PBS, the sections were stained with DAPI (catalog no. D1306, Thermo Fisher Scientific, MMAS, United States) for 10 min at room temperature and washed again with PBS. The sections were sealed with neutral resin and observed under a fluorescence microscope.

### Western Blot

Tissue samples of the cortex and hippocampus were lysed together in RIPA buffer (Beyotime, Shanghai, China). Supernatants were collected, and proteins were quantified using the BCA Protein Assay kit (Beyotime, Shanghai, China). Denatured protein samples were separated on 10% sodium dodecyl sulfate-polyacrylamide electrophoresis (SDS-PAGE) gels and then transferred to polyvinyl difluoridine (PVDF) membranes. Membranes were blocked with 5% non-fat dry milk for 2 h and incubated with the following primary antibodies overnight at 4°C: Bax (diluted 1:1000, catalog no. ab32503, Abcam, Cambridge, United Kingdom), Bcl2 (diluted 1:1000, catalog no. ab32124, Abcam, Cambridge, United Kingdom), cleaved caspase-3 (diluted 1:500, catalog no. ab13847, Abcam, Cambridge, United Kingdom), LC3B (diluted 1:1000, catalog no. ab48394, Abcam, Cambridge, United Kingdom), Beclin 1 (diluted 1:2000, catalog no. ab207612, Abcam, Cambridge, United Kingdom), AKT (diluted 1:500, catalog no. ab8805, Abcam, Cambridge, United Kingdom), mTOR (diluted 1:2000, catalog no. ab2732, Abcam, Cambridge, United Kingdom), S6K1 (diluted 1:5000, catalog no. ab32529, Abcam, Cambridge, United Kingdom), 4E-BP1 (diluted 1:1000, catalog no. ab2606, Abcam, Cambridge, United Kingdom), or GAPDH (diluted 1:5000, catalog no. ab8245, Abcam, Cambridge, United Kingdom). The membranes were then incubated with goat anti-rabbit IgG H&L (HRP) (diluted 1:5000, catalog no. ab6721, Abcam, Cambridge, United Kingdom) or goat anti-mouse IgG H&L (HRP) (diluted 1:5000, catalog no. ab205719, Abcam, Cambridge, United Kingdom) secondary antibody for 2 h at room temperature. Protein bands were visualized using an enhanced chemiluminescence (ECL) kit (catalog no. 35050, Pierce, MMAS, United States) and quantified by scanning densitometry using ImageJ software.

### Statistical Analysis

All of the statistical analyses were performed using SPSS 19.0 software. The data are expressed as mean ± standard deviation. Comparisons between groups were performed using one-way analysis of variance. Intra-group comparisons were performed using a repeated measures design. Values of *p* < 0.05 were considered statistically significant.

## Results

### Resveratrol Significantly Decreased Neurological Deficit Scores in CCH Rats

**FIGURE 2:**
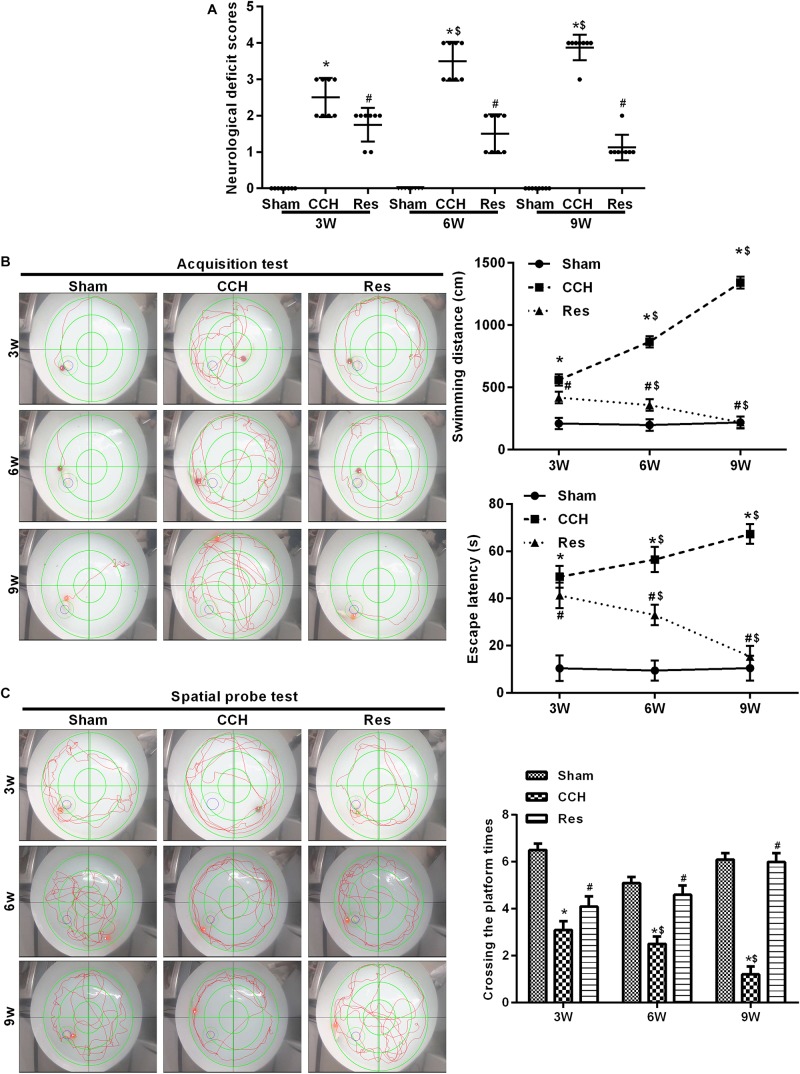
Resveratrol improved neurological damage and cognitive function in CCH rats. Neurological impairment was measured by the Bederson scoring method. Memory performance was measured in the Morris water maze test. **(A)** Neurological deficit scores. **(B,C)** Morris water maze test. ^∗^*p* < 0.05, vs. Sham group; ^#^*p* < 0.05, vs. CCH group; ^$^*p* < 0.05, vs. 3 weeks. Sham group, treated with an equal volume of vehicle; CCH group, chronic cerebral hypoperfusion and no treatment; CCH + Res group, CCH and treated with resveratrol.

We first examined the effect of CCH on untreated rat brains. Neurological deficit scores increased after BCCAO, and both spatial cognitive ability and spatial memory decreased in CCH rats compared with the Sham group (*p* < 0.05). Neurological deficit scores gradually increased 3, 6, and 9 weeks after ligation. Repeated resveratrol treatment decreased neurological deficit scores in rats (Figure 2A). We then tested memory performance in the Morris water maze test (Figures 2B,C). Three weeks after BCCAO, the escape latency and swimming path significantly increased in CCH rats compared with the Sham group (*p* < 0.05), with more pronounced effects at 6 and 9 weeks of ischemia (*p* < 0.05, 9 weeks vs. 3 weeks).

### Resveratrol Alleviated Brain Damage in CCH Rats

**FIGURE 3:**
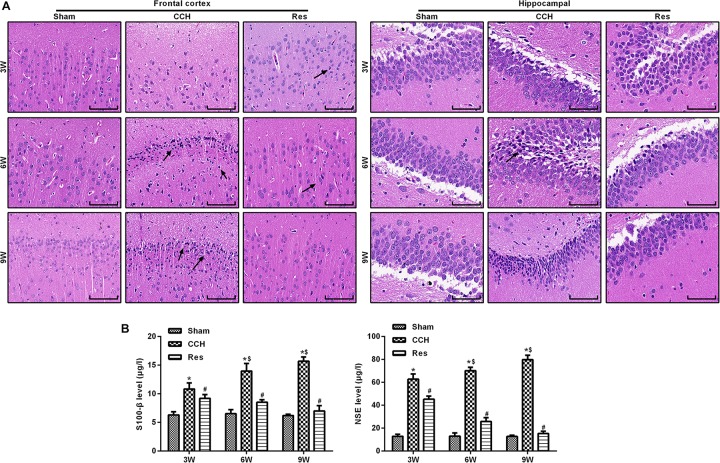
Resveratrol alleviated brain damage in CCH rats. Neuronal pathological changes were detected by HE staining in the frontal cortex and hippocampal CA1 area in each group. ELISA was used to observe the brain damage markers S-100β and NSE. **(A)** Hematoxylin and eosin staining. Scale bar = 50 μm. **(B)** S-100β and NSE levels were detected by ELISA. ^∗^*p* < 0.05, vs. control group; ^#^*p* < 0.05, vs. CCH group; ^∗^*p* < 0.05, vs. Sham group; ^#^*p* < 0.05, vs. CCH group; ^$^*p* < 0.05, vs. 3 weeks. Sham group, treated with an equal volume of vehicle; CCH group, chronic cerebral hypoperfusion and no treatment; Res group, CCH and treated with resveratrol.

The retinal cortex and hippocampal CA1 area were monitored in CCH rats that were treated with resveratrol (Res group) at 3, 6, and 9 weeks of ischemia. Ischemia that was induced by CCH gradually improved, and nuclei were large and round with clear nucleoli, indicating that resveratrol improved brain damage that was caused by CCH (Figure 3A). Compared with the Sham group, levels of the brain damage markers S-100β and NSE gradually increased in the CCH group at 3, 6, and 9 weeks of ischemia, whereas S-100β and NSE levels significantly decreased in the Res group at the corresponding time points compared with the CCH group (*p* < 0.05), suggesting that resveratrol alleviated brain damage in CCH rats (Figure 3B).

**FIGURE 4:**
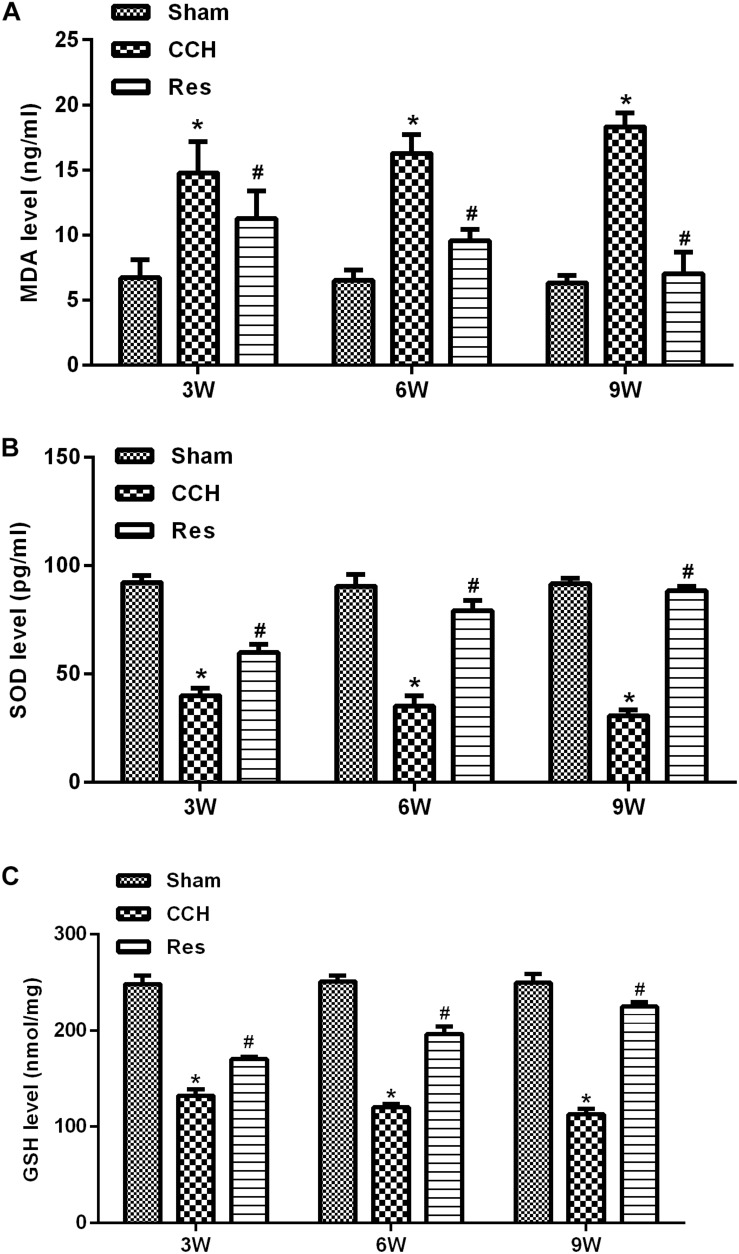
Resveratrol inhibited oxidative stress in CCH rats. ELISA was used to detect oxidative stress factors. **(A)** Levels of MDA. **(B)** Levels of SOD. **(C)** Levels of GSH. ^∗^*p* < 0.05, vs. Sham group; ^#^*p* < 0.05, vs. CCH group. Sham group, treated with an equal volume of vehicle; CCH group, chronic cerebral hypoperfusion and no treatment; Res group, CCH and treated with resveratrol.

**FIGURE 5:**
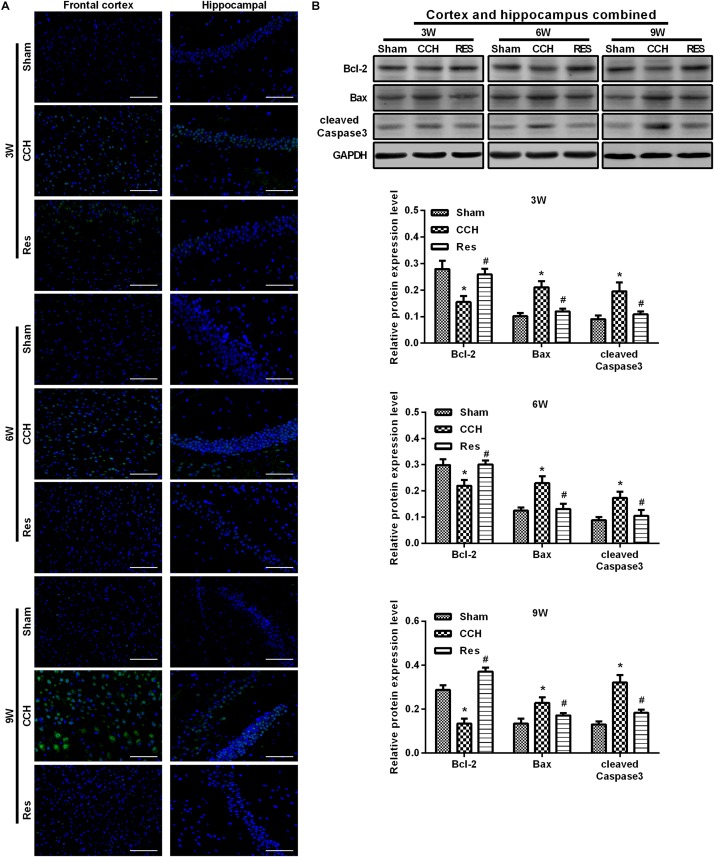
Resveratrol inhibited neuronal apoptosis in CCH rats. TUNEL staining was used to detect neuronal apoptosis in the frontal cortex and hippocampal CA1 area in each group. Apoptosis-related proteins were detected by Western blot. **(A)** TUNEL staining. Scale bar = 50 μm. **(B)** Western blot. ^∗^*p* < 0.05, vs. Sham group; ^#^*p* < 0.05, vs. CCH group. Sham group, treated with an equal volume of vehicle; CCH group, chronic cerebral hypoperfusion and no treatment; Res group, CCH and treated with resveratrol.

To evaluate oxidative stress between experimental groups, we performed ELISAs for several known markers of oxidative stress. After resveratrol treatment, MDA levels gradually decreased in the frontal cortex and hippocampal CA1 area in the Res group, whereas SOD and GSH levels gradually increased (Figure 4). Rats that were subjected to CCH exhibited apparent neuronal apoptosis. The rate of apoptosis gradually increased over time and reached its highest point at 9 weeks of ischemia (Figure 5A). Bcl-2 expression gradually decreased, whereas cleaved caspase-3 and Bax increased in CCH rats compared with the Sham group (*p* < 0.05) at later timepoints. Repeated resveratrol treatment significantly improved neuronal apoptosis, increased Bcl-2 expression, and decreased cleaved caspase-3 and Bax expression compared with the CCH group (*p* < 0.05; Figure 5B).

### Resveratrol Protected Against CCH via the PI3K/AKT/mTOR Pathway

**FIGURE 6:**
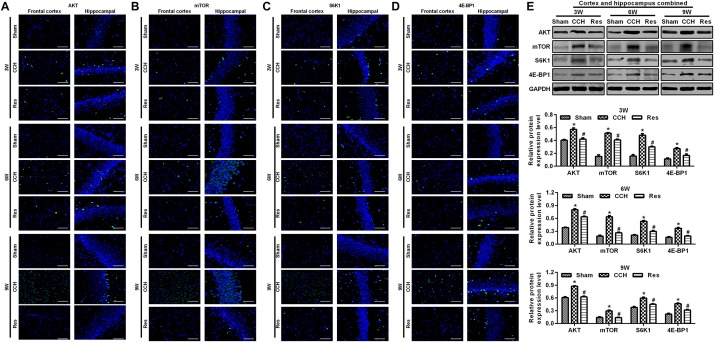
Resveratrol reduced brain damage in CCH rats via the AKT/mTOR signaling pathway. Immunofluorescence staining and Western blot were used to detect the expression of AKT/mTOR signaling pathway-related proteins. **(A–D)** Immunofluorescence staining. Scale bar = 50 μm. **(E)** Western blot. ^∗^*p* < 0.05, vs. Sham group; ^#^*p* < 0.05, vs. CCH group. Sham group, treated with an equal volume of vehicle; CCH group, chronic cerebral hypoperfusion and no treatment; Res group, CCH and treated with resveratrol.

**FIGURE 7:**
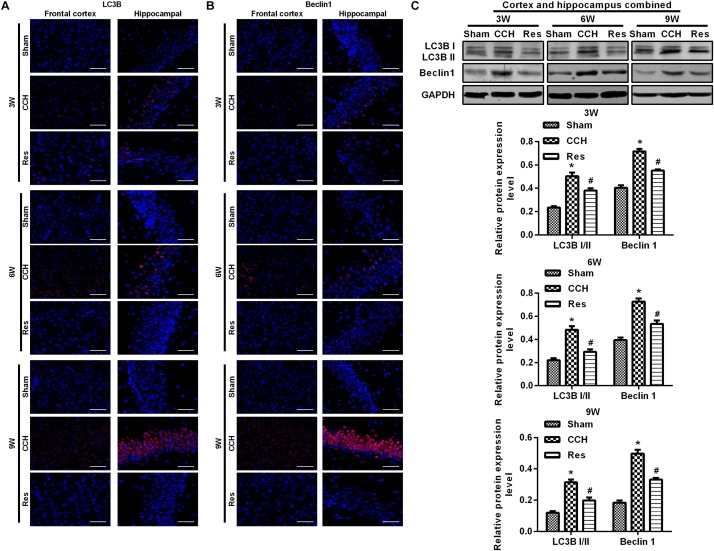
Resveratrol regulated autophagy in CCH rats. Immunofluorescence staining and Western blot were used to detect the expression of LC3B and Beclin 1. **(A,B)** Immunofluorescence staining. Scale bar = 50 μm. **(C)** Western blot. ^∗^*p* < 0.05, vs. Sham group; ^#^*p* < 0.05, vs. CCH group. Sham group, treated with an equal volume of vehicle; CCH group, chronic cerebral hypoperfusion and no treatment; Res group, CCH and treated with resveratrol.

To explore the possible mechanism by which these physiological changes occurred, we examined the AKT/mTOR signaling pathway in each experimental group. The expression of AKT, mTOR, S6K1, and 4E-BP1 in the frontal cortex and hippocampal CA1 area increased over time in the CCH group compared with the Sham group (*p* < 0.05; Figures 6A–D). After resveratrol treatment for 3, 6, and 9 weeks, the expression of AKT, mTOR, S6K1, and 4E-BP1 gradually decreased in the frontal cortex and hippocampal CA1 area compared with the CCH group (*p* < 0.05; Figure 6E). The AKT/mTOR signaling pathway has been linked to autophagy (Heras-Sandoval et al., 2014). Therefore, we evaluated markers of autophagy in the different experimental groups. At extended ligation times, rats in the CCH group exhibited higher LC3B and Beclin 1 expression in the frontal cortex and hippocampal CA1 area compared with the Sham group (*p* < 0.05; Figures 7A,B). After resveratrol treatment, the expression of LC3B and Beclin 1 gradually decreased compared with the CCH group (*p* < 0.05; Figure 7C).

### The Protective Effect of Resveratrol Against CCH Was Blocked by a PI3K Inhibitor

**FIGURE 8:**
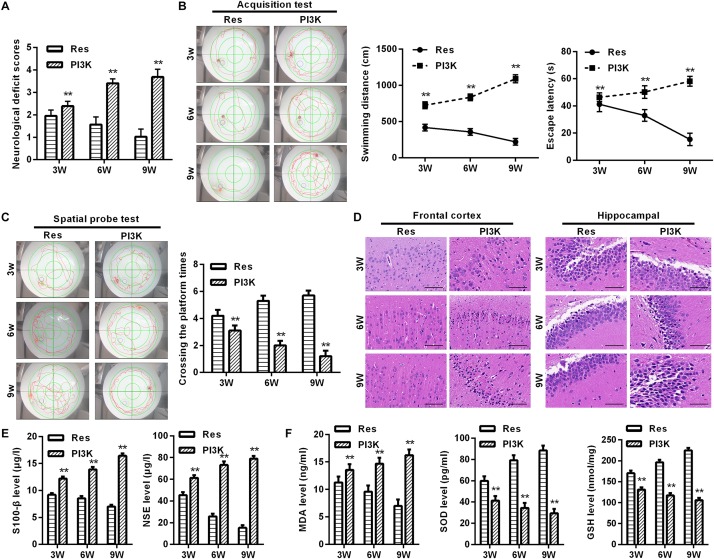
PI3K inhibition reversed the therapeutic effects of resveratrol in CCH rats. Neurological impairment was measured by the Bederson scoring method. Memory performance was measured in the Morris water maze test. Neuronal pathological changes were detected by HE staining in the frontal cortex and hippocampal CA1 area in each group. ELISA was used to observe markers of brain damage and oxidative stress. **(A)** Neurological deficit scores. **(B,C)** Morris water maze test. **(D)** Hematoxylin staining. Scale bar = 50 μm. **(E)** Levels of S-100β and NSE. **(F)** Levels of MDA, SOD, and GSH. ^∗∗^*p* < 0.05, vs. Res group. Res group, CCH and treated with resveratrol; PI3K group, CCH and treated with resveratrol and the PI3K inhibitor LY294002.

**FIGURE 9:**
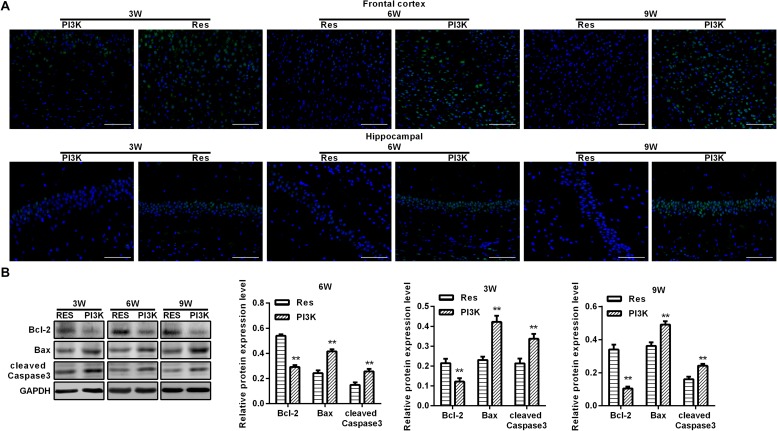
PI3K inhibition reversed the therapeutic effects of resveratrol in CCH rats. TUNEL assays were performed to evaluate neuronal apoptosis in the frontal cortex and hippocampal CA1 area in each group. Apoptosis-related proteins were detected by Western blot. **(A)** TUNEL staining. Scale bar = 50 μm. **(B)** Western blot. ^∗∗^*p* < 0.05, vs. Res group. Res group, CCH and treated with resveratrol; PI3K group, CCH and treated with resveratrol and the PI3K inhibitor LY294002.

**FIGURE 10:**
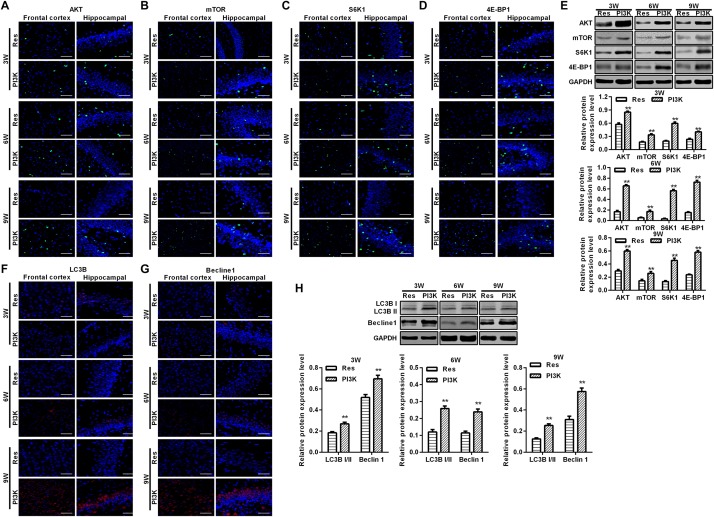
PI3K inhibition reversed the therapeutic effects of resveratrol in CCH rats. Immunofluorescence staining and Western blot were used to detect expression of the AKT/mTOR signaling pathway-related proteins LC3B and Beclin 1. **(A–D,F,G)** Immunofluorescence staining. Scale bar = 50 μm. **(E,H)** Western blot. ^∗∗^*p* < 0.05, vs. Res group. Res group, CCH and treated with resveratrol; PI3K group, CCH and treated with resveratrol and the PI3K inhibitor LY294002.

To evaluate whether the protective effects of resveratrol against CCH depend on the PI3K/AKT/mTOR pathway, we treated CCH rats with both the PI3K inhibitor LY294002 and resveratrol. Neurological deficit scores in the PI3K group gradually increased, and their cognitive and spatial memory abilities were poor compared with the Res group (*p* < 0.05; Figures 8A–C). Brain tissue in the frontal cortex and hippocampal CA1 area was decrease in the PI3K group, and the hippocampus was atrophied. Neuronal injury was most apparent at 9 weeks of ischemia, with an increase in bubble-like structures in the PI3K group compared with the Res group (*p* < 0.05; Figure 8D). The expression of MDA, S-100β, and NSE gradually increased with administration time in the PI3K group compared with the Res group, whereas SOD and GSH expression gradually decreased (*p* < 0.05; Figures 8E,F). The rate of neuronal apoptosis in the frontal cortex and hippocampal CA1 area increased in the PI3K group, reaching its highest point at 9 weeks (Figure 9A). Bcl-2 expression decreased over time in the PI3K group compared with the Res group, and the expression of cleaved caspase-3 and Bax gradually increased (*p* < 0.05; Figure 9B). The expression of AKT, mTOR, S6K1, 4E-BP1, LC3B, and Beclin 1 increased in the frontal cortex and hippocampal CA1 area in the PI3K group with prolonged administration compared with the Res group (*p* < 0.05; Figure 10). Future studies will investigate the detailed mechanism of action of resveratrol, including its targeted proteins.

## Discussion

Chronic cerebral hypoperfusion is a pathological disorder that is known to lead to cognitive dysfunction. To investigate the effect of resveratrol on cognitive function in CCH, a rat model of CCH was established by permanently ligating the bilateral common carotid arteries. After establishing the CCH model, resveratrol was administered for 21 consecutive days. Cognitive function was evaluated in rats in the Morris water maze at 3, 6, and 9 weeks of ischemia. Cognitive function improved in CCH rats after resveratrol administration. We examined pathological damage to the frontal cortex and hippocampus using HE staining and found decreases in markers of oxidative stress and brain injury following resveratrol treatment. The Western blot data showed that resveratrol activated autophagy, decreased LC3B and Beclin 1 expression, and inhibited the expression of AKT/mTOR signaling pathway-related proteins. These results were the most significant at 9 weeks. After the administration of a PI3K inhibitor, the expression of AKT/mTOR signaling pathway-related proteins increased, and autophagy decreased, indicating that the neuroprotective effects of resveratrol were inhibited. However, we did not use quantitative polymerase chain reaction to detect the transcription levels of mTOR-related genes. Thus, we were unable to determine whether resveratrol exerts its functions through transcriptional regulation or an increase in the half-life of mTOR-related proteins. Future studies will seek to unveil the molecular mechanism of action of resveratrol. Previous studies have shown that neuronal cells in the frontal cortex and hippocampus play important roles in maintaining normal learning and cognitive ability. Therefore, blocking neuronal apoptosis in the frontal cortex and hippocampus is important for improving cognitive dysfunction. The present study showed that resveratrol improved cognitive function in CCH rats and improved neuronal damage in the frontal cortex and hippocampus.

Resveratrol is a non-flavonoid polyphenolic compound that is produced by plants (Pezzuto, 2018). Recent studies reported that polyphenols exert protective effects on many aging-related central nervous system diseases, and the neuroprotective effects of resveratrol have also been confirmed in many animal models (Zhao et al., 2017; Palle and Neerati, 2018). Ghanim et al. (2010) found that a resveratrol extract inhibited ROS production, and Martinez and Moreno (2000) found that resveratrol exerted a strong inhibitory effect on superoxide radicals, indicating that resveratrol can reduce or prevent the occurrence of oxidative stress. Neuronal degeneration that is caused by CCH is a pathological basis for neuronal apoptosis (Jing et al., 2015). Many studies have shown that resveratrol can alleviate axonal degeneration and inhibit hippocampal neuronal apoptosis (Moriya et al., 2011; Zhang et al., 2015; Tian et al., 2016). Resveratrol was shown to protect the nervous system by regulating autophagy and clearing pathological proteins, thus contributing to the treatment of neurological diseases, but its mechanism of action is still unclear (Hu et al., 2017). The present study established a rat model of CCH to explore the mechanism of its neuroprotective effects. Our results showed that CCH rats exhibited the gradual recovery of cognitive and learning abilities, coinciding with improvements in pathological damage in the frontal cortex and hippocampal CA1 area with prolonged resveratrol administration. The present study focused on promoting cognitive function. We did not evaluate changes in dendritic spines. Furthermore, factors that are indicative of oxidative stress and apoptosis gradually decreased with resveratrol treatment, with decreases in AKT, mTOR, S6K1, and 4E-BP1 levels and inhibition of the expression of LC3B and Beclin 1. These findings indicate that resveratrol improves brain tissue damage in the frontal cortex and hippocampal CA1 area by inhibiting oxidative stress, thereby attenuating neuronal apoptosis. This was associated with decreases in the expression of AKT/mTOR signaling pathway-associated proteins and increases in autophagy in the frontal cortex and hippocampal CA1 region.

The AKT/mTOR signaling pathway is a classic anti-apoptosis pathway that is involved in various cellular activities and closely related to cell survival, the cell cycle, cell proliferation, and apoptosis (Wang et al., 2017; Zhang et al., 2018). Several recent studies have shown that regulating this pathway can prevent or improve neurodegenerative diseases (Guillot et al., 2016; Giacoppo et al., 2017). Resveratrol appears to regulate the cell cycle through numerous molecular mechanisms (Medina-Aguilar et al., 2016; He Y. et al., 2017; Singh et al., 2017). In T-ALL cells, resveratrol inhibited the expression of AKT, mTOR, and 4E-BP1 and activated p38 and mitogen-activated protein kinase signals to induce apoptosis (Ge et al., 2013). In addition to controlling the rate of aging of cells and tissues, mTOR signaling is also important for inhibiting autophagy and the formation of lysosomes (Yu et al., 2010). Numerous studies have shown that there may be a common regulatory factor between autophagy and apoptosis. In the model of focal cerebral ischemia and ischemic injury, Beclin 1 may be involved in the activation of caspase-3, and the anti-apoptotic protein Bcl-2 binds to Beclin 1 to block autophagy (Ciechomska et al., 2009; Yang et al., 2017; Ding et al., 2018; Wang J. et al., 2018). In the present study, we established a rat model of CCH and took samples at different timepoints of resveratrol treatment. Our results indicated that resveratrol exerted an inhibitory effect on neuronal apoptosis by increasing the expression of Bcl-2, decreasing the expression of cleaved caspase-3 and Bax, and downregulating the expression of AKT, mTOR, S6K1, and 4E-BP1 in brain tissue. These resveratrol-induced changes were reversed by the PI3K inhibitor LY294002. The present results suggest that resveratrol may activate autophagy via the AKT/mTOR pathway to improve neuronal apoptosis in CCH rats. Thus, inhibiting the AKT/mTOR signaling pathway-mediated activation of autophagy may be important for the treatment of cognitive decline.

## Conclusion

Resveratrol improved pathological damage and cognitive function in a rat model of CCH by activating autophagy to reduce oxidative stress and inhibiting neuronal apoptosis in the frontal cortex and hippocampus. These beneficial effects of resveratrol may be regulated by the AKT/mTOR signaling pathway.

## Data Availability

The raw data supporting the conclusions of this manuscript will be made available by the authors, without undue reservation, to any qualified researcher.

## Ethics Statement

All of the animal experiments conformed to internationally accepted standards and were approved by the Experimental Animal Welfare and Ethics Committee of China Medical University (IACUC No. 2018097).

## Author Contributions

NW and ZX conceived and designed the experiments. NW, JH, CP, JW, MM, and XS performed the experiments. JW, MM, and XS analyzed the data. NW and ZX wrote the manuscript.

## Conflict of Interest Statement

The authors declare that the research was conducted in the absence of any commercial or financial relationships that could be construed as a potential conflict of interest.
